# *Acinetobacter baumannii *in intensive care unit: A novel system to study clonal relationship among the isolates

**DOI:** 10.1186/1471-2334-8-79

**Published:** 2008-06-08

**Authors:** Carla Fontana, Marco Favaro, Silvia Minelli, Maria Cristina Bossa, Gian Piero Testore, Francesca Leonardis, Silvia Natoli, Cartesio Favalli

**Affiliations:** 1 Department of Experimental Medicine and Biochemical Sciences, "Tor Vergata" University of Rome, Via Montpellier 1, 00133 Rome, Italy; 2 Clinical Microbiology Laboratories, Polyclinic of Tor Vergata, Viale Oxford 81, 00133 Rome, Italy; 3 Public Health Department, Infectious Diseases, Polyclinic of Tor Vergata, Viale Oxford 81, 00133 Rome, Italy; 4 Department of Surgery, Intensive Care Unit – Polyclinic of Tor Vergata, Viale Oxford 81, 00133 Rome, Italy

## Abstract

**Background:**

The nosocomial infections surveillance system must be strongly effective especially in highly critic areas, such as Intensive Care Units (ICU). These areas are frequently an epidemiological epicentre for transmission of multi-resistant pathogens, like *Acinetobacter baumannii*. As an epidemic outbreak occurs it is very important to confirm or exclude the genetic relationship among the isolates in a short time. There are several molecular typing systems used with this aim. The Repetitive sequence-based PCR (REP-PCR) has been recognized as an effective method and it was recently adapted to an automated format known as the DiversiLab system.

**Methods:**

In the present study we have evaluated the combination of a newly introduced software package for the control of hospital infection (VIGI@ct) with the DiversiLab system. In order to evaluate the reliability of the DiversiLab its results were also compared with those obtained using f-AFLP.

**Results:**

The combination of VIGI@ct and DiversiLab enabled an earlier identification of an *A. baumannii *epidemic cluster, through the confirmation of the genetic relationship among the isolates. This cluster regards 56 multi-drug-resistant *A. baumannii *isolates from several specimens collected from 13 different patients admitted to the ICU in a ten month period. The *A. baumannii *isolates were clonally related being their similarity included between 97 and 100%. The results of the DiversiLab were confirmed by f-AFLP analysis.

**Conclusion:**

The early identification of the outbreak has led to the prompt application of operative procedures and precautions to avoid the spread of pathogen. To date, 6 months after the last *A. baumannii *isolate, no other related case has been identified.

## Background

Interest in *Acinetobacter baumannii *continues to rise. One of the main reasons is the emergence of multi-resistant strains, which cause sudden outbreaks of infection involving several patients in a ward and/or in different areas of the hospital [1,2]. In clinical practice, *Acinetobacter *infections are influenced by various risk-factors: the use of medical devices (such as: endotracheal tubes, intravascular and urinary catheters), the exposure to broad-spectrum antibiotics and the type of ward where a patient is admitted (e.g. in ICUs the infection rate is often high) [3,4]. The sum of these factors contributes to sudden outbreaks that are difficult to control and prevent. Another worrisome aspect of *A. baumannii *infections is that the patients with symptoms are likely to represent the tip of the colonization iceberg, because significant unobserved dissemination also occurs [1]. Ideally, the diagnostic laboratory should be involved in active as well as in passive (or laboratory-based) surveillance, and in the subsequent molecular typing of microbial pathogens involved in an epidemic cluster [5-7]. Microbial typing methods have become an integral part of clinical laboratories. Many microbial typing methods are available: PCR multilocus enzyme electrophoresis (MLEE), moltilocus sequence typing (MLST), pulsed-field gel electrophoresis (PFGE), restriction fragment length polymorphisms (RFLP), DNA sequencing, ribotyping, restriction fragment length polymorphism studies, randomly amplified polymorphism DNA (RAPD), amplified fragment length polymorphism (AFLP) and repetitive sequence-based PCR (REP-PCR) [8]. The choice of the proper typing method is not easy and limitations are described for each: MLEE is useful only to provide the overall genetic relatedness and diversity; AFLP yield complex DNA profile that can be challenging to interpret; RAPD has poor inter- and intra-laboratory reproducibility; MLST are labour intensive and costly; PFGE has difficulty in resolving bands of similar size. Essentially very few typing methods assess outbreaks in real time, provide comprehensive surveillance and epidemiological data, and have data-archiving capability to build libraries. The REP-PCR method uses primers that target non-coding repetitive sequences interspersed throughout the bacterial DNA and is an established approach for subspecies classification. It was recently commercially adapted to an automated format the DiversiLab, which allows simplistic data elaboration, archiving, retrieval and reporting [8]. In the present paper we show the results of our experience, in studying an *A. bauamannii *outbreak, based on the combined use of a laboratory-based surveillance system (the VIGI@ct) and a new molecular typing system: the DiversiLab. In order to validate the system the results of the DiversiLab were compared with those obtained by using f-AFLP assay.

## Methods

### Laboratory-based surveillance system

The Microbiology laboratory of "Tor Vergata" Teaching Hospital uses a surveillance system named VIGI@ct^®^, version 1.2 (bioMèrieux; Las Balmas, France). The system allows the monitoring of pathogen circulation either those with multi-resistant phenotypes (Multi Drug Resistant) or those defined as alert organisms *in sensu lato*. In our recent publication we have already extensively described how efficiently the system can be used to control, in real time, the hospital infection as well as the diffusion of pathogens [9]. The VIGI@ct is designed to work with Windows NT and is connected to the Laboratory Information System (LIS; Dasilab-Delphi; Dasit) so that the VIGI@ct receives all the analysis requested from the clinicians in the wards and collects them in the LIS. This connection is also used to acquire additional patient information such as admission and discharge dates or internal location changes, as well as demographic data supplied by the LIS. The LIS is also linked with the automated system VITEK 2 (bioMérieux) used in our laboratory to perform microbial identification as well as the antimicrobial susceptibility testing of the isolates. Every day the microbiologists launch the data integration from the LIS and VIGI@ct using two program functions: 'receiving the requests' and 'receiving the results' from the LIS. In this manner, the VIGI@ct receives all the bacteriological data coming from the LIS connection which are concentrated in the general bacteriology database of the VIGI@ct, according to the user-defined criteria, where they can be viewed or used at any time for epidemiological studies. This type of connection allows the VIGI@ct real time identification of all pathogens responsible for hospital acquired infections (HAIs) using selection criteria established by the user and monitored continuously by the program. To define an HAIs the CDC criteria are used.

### Patients

The thirteen patients (nine males and four females) had a median age of 59; Seven patients displayed a simple colonization (based on clinical evidence), while the remaining six developed infection (Table 1).

**Table 1:** *A. baumannii*: samples and first collection date by each patient.

**Patients**	**First sample collection date**	**Specimen (no. of examined specimens)**	**Infection/colonization**	**Sub clone no.**
Patient 1	03-December-2005	CVC (2), Urine (2); ASB (2)	Infection	**1**
Patient 2	18-October-2005	BAL (2), Urine (1), CVC (2), Blood (2)	Infection	**2**
Patient 3	16-December-2005	ASB (2)	Colonization	**3**
Patient 4	21-December-2005	ASB (1), Blood (6),	Infection	**4**
Patient 5	11-January-2006	Urine (1)	Colonization	**5**
Patient 6	05-May-2006	ASB (2)	Colonization	**6**
Patient 7	08-May-2006	Blood (3), ASB (1), Urine (5)	Infection	**7**
Patient 8	18-June-2006	ASB(1), CVC(1), Blood(5)	Infection	**8**
Patient 9	22-June-2006	ASB(3), Blood(2)	Infection	**9**
Patient 10	12-July-2006	Stool(1)	Colonization	**10**
Patient 11	22-July-2006	ASB(3), BAL(1), CVC(2) SW(1)	Colonization	**11**
Patient 12	26-July-2006	Urine(1)	Colonization	**12**
Patient 13	28-July-2006	ASB(1)	Colonization	**13**

ASB: endothracheal aspirates, BAL:bronchoalveoar lavage, CVC: central venous chateter, SW: swab wound.

### *A. baumannii *clinical isolates

From October 2005 to August 2006, 56 isolates of *A. baumannii *(41 isolated in infected patients and 15 in colonized patients), from 13 patients admitted to the medical ICU, were evidenced by the VIGI@ct^®^. Table 1 lists the sample data collected from each patient. All *A. baumannii *isolates were biochemically characterized and studied for their antimicrobial susceptibilities using VITEK 2 (bioMérieux). After isolation the strains were stored at -70°C in 0.5 ml of defibrinated bovine blood for further investigation.

### Clones

Clones are identified by very high levels of similarity. The latter is calculated using DiversiLab system by Pearson correlation coefficient or by Dice coefficient for f-AFLP [8-10].

### Environmental screening

The Hospital Infection Control team of our hospital (HIC; composed of a microbiologist, a hygienist, a chemist, an expert in infectious disease and several hospital managers) decided to monitor the presence of the micro-organism in the ICU environment. Environmental sampling was carried out in the ICU at the end of July. A total of 168 sites were sampled from the patients' immediate environment. These included patients' monitors, bed frames, X-ray illuminator, echograph, equipment trolley, and any other surface, including desktops of the clinicians, as well as walls and floor near the patient's beds. Each site was catalogued and the same 168 sites were investigated again after one month (after the intensive cleaning of the ward). The sites were sampled using sterile swabs moistened with sterile saline solution and then processed as reported by Denton *et al*. [11].

### Carbapenem resistance characterization

PCR reaction for detection of the *bla*_IMP _and *bla*_VIM _genes was performed according to Docquier *et al*; the *bla*_OXA-58 _was investigated by PCR assays on genomic DNAs using previously described primers pairs [12,13].

### Fingerprinting of the isolates using the DiversiLab system

The isolates were fingerprinted using the REP-PCR automated in the DiversiLab system (Bacterial Bar Codes, Inc; now distributed by bioMèrieux), version 3.3. DNA was extracted from a bacterial suspension in physiologic saline, with a turbidity 0.5 (Mac Farland scale) using the UltraClean Microbial DNA Isolation kit (MO BIO Laboratories, Inc.) according to the manufacturer's instructions. After extraction the DNA was electrophoresed in 1.5% agarose and stained with Sybrsafe (Invitrogen). The fingerprinting of the isolates was performed using *Acinetobacter *Kit (DiversiLab) following the instructions contained in "REP-PCR worksheet". At the end of the amplification reaction, samples were loaded in a chip (following the loading sequence numbers illustrated in the chip Worksheet) and run using the Agilent 2100 Bioanalyzer (Agilent Technologies). When the run was completed the results were sent via internet to the Bacterial Barcodes Database and analysed by Bionumerics software. Analysis was performed using the DiversiLab software with the Pearson correlation coefficient to determine the distance matrices and the un-weighted-pair group method with arithmetic mean (UPGMA) to create dendrograms. Report were automatically generated and included: dendrogram, electropherograms, virtual gel images, similarity matrix, scatter plots, and selectable demographic fields to aid the interpretation of the data [8,11,12,14].

### Fingerprinting of the isolates using f-Amplified Fragment Length Polymorphism

The genetic relationship among the isolates was also determined using the commercial kit f-AFLP Microbial fingerprinting (Applera; Foster City, California) according to the manufacturer's instructions. The f-AFLP reactions were loaded and run on the ABI 310 DNA genetic analyzer (Applera). Each f-AFLP reaction was analysed using Genescan software and Genographer program version 1.6.0 (kindly provided by James J. Benham). Cluster analysis was performed using the UPGMA. The percentage similarity between patterns was calculated using the Dice correlation coefficient. The cluster analysis of a single outbreak was completed in a time ranging from 72 h to 96 h [9].

### Document Ethical approval

n°52/08, by Ethical Committee (Prof P. Fucci)

## Results

### Description of the outbreak

From October 2005 to August 2006, 56 clinical isolates of *A. baumannii *were evidenced by the VIGI@ct^®^. The micro-organism first appeared in October 2005, and from that time it was responsible of an epidemic cluster consisting of 56 strains isolated from 13 patients admitted to the medical ICU. Our medical ICU is a seven-bed unit (five in an open-plan area and two in single rooms), in which are admitted 135 patients per year. The frequency of HAI ranges from 20 to 43%.

The ten most frequent pathogens isolated in the ICU, from October 2005 and October 2006 were: *Pseudomonas aeruginosa *(16%), *Klebsiella pneumoniae *(15%), *Staphylococcus epidermidis *(9%), *Enterococcus faecalis *(8%), *Escherichia coli *(7%), *Staphylococcus aureus *(7%), *A. baumannii *(7%), *Candida albicans *(7%), *Enterococcus faecium *(3%), *Staphylococcus haemolyticus *(2.5%).

The first patient, in whom *A. baumannii *was isolated, was admitted to the ICU coming from another hospital (in October). This patient, evidently imported the *A. baumannii *to our ICU ward, where the pathogen was cross-transmitted to other patients. Out of 13 patients, seven displayed simple colonization (based on clinical evidence) while the others developed infections (Table 1). In January the outbreak became evident and to reduce infection rate the environmental cleaning was enhanced and the hand hygiene procedure was re-enforced (providing staff with alcohol hand rub). Initially these measures appeared to be effective, but the pathogen diffusion re-started on May. In July, in two weeks, the *A. baumannii *was isolated in four different patients. The laboratory promptly documented the epidemic peak and the head of ICU prepared a detailed report for the Sanitary Direction (SD) describing the last infection cases. The SD decided to close the ICU for about one month and existing patients were moved to the ICUs of adjacent hospitals. The ward environment was thoroughly cleaned using detergent and disinfectant and all disposable materials were replaced. Since the ward was re-opened, *A. baumannii *has no longer been isolated.

### Clinical isolates

**Figure 1:**
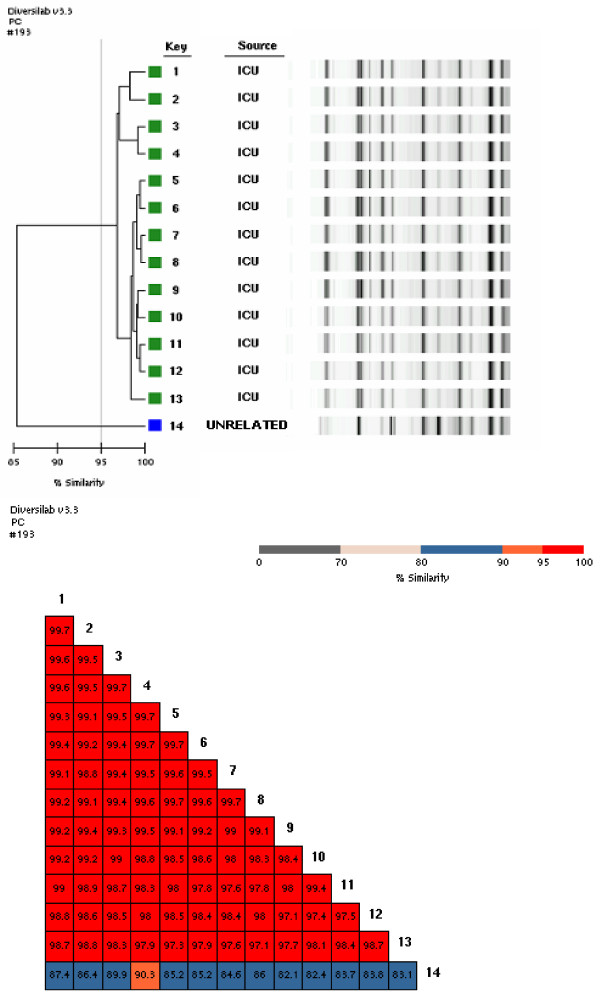
**Dendrogram and similarity matrix for *A. baumannii *sub clones from 13 patients**. 1–13 = sub clones from 13 patients. 14 = an unrelated strain of *A. baumannii*, not from ICU patients.

Fifty-six isolates of *A. baumannii *obtained from 13 patients, were cultured from a variety of sites, including endothracheal aspirates (ASB), bronchoalveoar lavage (BAL), central venous device tips (CVC), blood, wound swab and urine samples (Table 1). Among the 56 isolates from 13 different patients we identified 13 sub clones. The results of the REP-PCR analysis (Pearson correlation coefficient) as well as that of f-AFLP (using the Dice's coefficient) demonstrate, in fact, that each patient was colonized/infected from a single sub clone (Table 1). To each sub clone was assigned a number that was the same used to identify the patient. Figure 1 reports the REP-PCR profiles of the 13 sub clones and their similarity matrix The REP-PCR analysis showed a clonal relationship among the isolates (being their similarity greater than the 97%).

All the clinical isolates had the same antibiograms, being susceptible to gentamicin and colistin, but resistant to imipimen, meropenem, 3^rd ^generation cephalosporins, amikacin, ciprofloxacin, ampicillin, aztreonam, and piperacillin-tazobactam. All strains tested were positive for the *bla*_OXA-58 _gene (data not shown).

### Environmental screening

**Figure 2:**
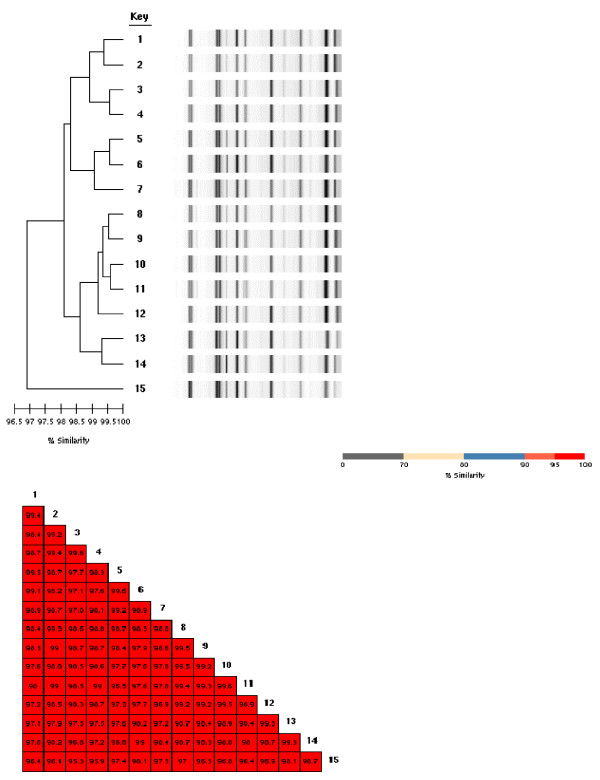
**REP-PCR profiles and similarity matrix of the environmental sub clones**. 1 = strain cultured from the ventilator of bed no.1; 2, 6, 11 = strain cultured from the floor near the patient beds no.1, 3,4 3, 9 = strain cultured from the desk surfaces of beds no.4 and 7; 4 = strain cultured from buttons on the ventilator of bed no.3; 5, 12 = strain cultured from ventilator keyboard of bed no.3 and 6; 7 = strain cultured from edge side of bed no.8; 8 = strain cultured from drawers of the bedside table near bed no.5; 10 = strain cultured from service desk near bed no.1; 13 = strain cultured from the floor at the entrance of ICU; 14 = strain cultured from folder of medical record of patient in the bed no.1. 15 = strain cultured from monitor keyboard of bed no.3.

Out of 168 environmental sites investigated, 15 were found to be contaminated by *A. baumannii*. Figure 2 shows the similarity matrix and the REP-PCR profiles of the environmental isolates, which are clonally related, as their similarity ranged from 97 to 100%. Following cleaning and disinfection of the ICU ward, no *A. baumannii *isolates were grown from repeated environmental cultures.

### Relationship among clinical and environmental isolates

**Figure 3:**
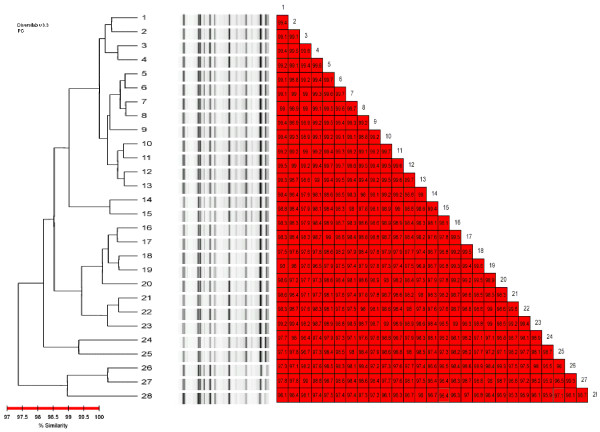
**Rep-PCR profiles of the environmental – as well as patients' sub clones**. 1 = patient's sub clone 9; 2 = environmental-sub clone1; 3 = environmental-sub clone 6; 4 = environmental-sub clone 5; 5 = patient's sub clone 5; 6 = patient's sub clone 6; 7 = patient's sub clone 7; 8 = patient's sub clone 8; 9 = environmental-sub clone 7; 10 = patient's sub clone 1; 11 = patient's sub clone 2; 12 = patient's sub clone 3; 13 = patient's sub clone 4; 14 = patient's sub clone 10; 15 = patient's sub clone 11; 16 = environmental-sub clone 9; 17 = environmental-sub clone 8; 18 = environmental-sub clone 10; 19 = environmental-sub clone 11; 20 = environmental-sub clone 12; 21 = environmental-sub clone 3; 22 = environmental-sub clone 4; 23 = environmental-sub clone 2; 24 = patient's sub clone 12; 25 = patient's sub clone 13; 26 = environmental-sub clone 13; 27 = environmental-sub clone 14; 28 = environmental-sub clone 15.

As shown in Figure 3, the genetic relationship is consistently high between the 15 environmental isolates (named sub clones) and the 13 sub clones from patients (the percent of similarity was greater than 97.5%) demonstrating transmission from patient-to-patient as well as spreading of the pathogen in the ICU environment.

### Comparison of REP-PCR and f-AFLP results

The results of f-AFLP assays (performed on patient – as well as on environmental-strains; data not shown) compared with those obtained using REP-PCR, showed to be very similar. Both exhibit high discriminatory power, but f-AFLP appeared to be more labour intensive than REP-PCR automated in the DiversiLab system. In fact, the cluster analysis using f-AFLP is completed in a time ranging from 72 h to 96 h, while the DiversiLab automation allows a complete microbial typing analysis in approximately 4 h.

## Discussion

The use of a combined method based on a laboratory surveillance system and molecular typing methods helped us in the rapid detection of pathogen circulation in our hospital [9]. The prompt identification of a pathogen, albeit not necessarily associated to an infection, is of great importance in such critical areas, like the ICUs, because they can lead to the immediate alert programme. The consequent application of precautionary measures can reduce the number of infected patients as well as of those colonized [11,15-17]. Close cooperation between clinicians and microbiologists is also important for resolving an epidemic event. Microbiologist plays a primary role in the study of isolates circulating in the hospital. Accomplishing this control requires adequate systems (hardware and software) for the real time control of pathogens dissemination. At the same time, the microbiologist must also be able to rapidly confirm or exclude the possibility of an epidemic cluster by performing fingerprinting of the isolates [4-6,9]. Electronic laboratory-based surveillance methods have already been demonstrated to be more useful than the detection of nosocomial infection by hospital-wide medical records, since the latter is neither as sensitive nor always consistently applied [18,19]. Also, our experience confirms the effectiveness of the use of surveillance software, such as VIGI@ct^®^, in the rapid and sensitive identification of HAIs. The VIGI@ct^® ^is a new system introduced for the control of HAIs, which is already used in several hospitals in Italy but our experience is the first to be described [9]. The combination of VIGI@ct^® ^and the DiversiLab system appeared to be strongly efficient for the early detection of pathogen circulation and in the consequent confirmation/exclusion of genetic relatedness among the isolates. These tools are particularly useful when the microbiologist is asked to analyze and compare a large number of isolates (from patients as well as the hospital environment) and needs a simple system to compare the genetic profiles of numerous isolates in a reasonable time. Our environmental investigation, revealing a consistent contamination, led to the closure of the ICU and to its decontamination by disinfection. The Sanitary Direction authorized the readmission of the patients in the ward only after a second environmental control revealed the eradication of the pathogen. The high cost of one month of ICU closure had a heavy consequence on the behaviour of the ICU staff, which was more careful in the application of prevention measures and protocols. To date no *A. baumannii *has been isolated from the environment, and although one recent patient admitted in the ICU was determined to be colonized by this pathogen, it remains a single isolate from a single patient, and no spread of the pathogen has occurred.

## Conclusion

The combination of REP-PCR and relative analysis of data in the DiversiLab seems to be an excellent choice. In our previous work we described the use of f-AFLP for the fingerprinting of the isolates, but although this technique appears to be good, particularly for its discriminatory power, it remains defective because of inadequate support from software that is unable to compare the fingerprinting of numerous isolates at the same time (it needs the use of an additional software). On the contrary the DiversiLab system has been proved to be easy to use. Our findings show that the DiversiLab results are reliable (fingerprinting analysis of the isolates obtained using the DiversiLab are similar to those obtained using f-AFLP). The system exhibits high levels of reproducibility, offers standardized kits and it is cost effective (the costs of the system has been already demonstrated to be favourable especially when compared to other methods, including f-AFLP) [8]. Moreover, the high level of automation of the system satisfies the needs of the laboratory desiring a more convenient, user-friendly surveillance system. The short time with which the microbiologist identifies a possible epidemic cluster and then confirms the genetic relationship among the isolates is extremely important because it allows the HIC of the hospital to promptly implement measures to stop the spread of a pathogen.

## List of abbreviations

ICU: Intensive Care Units, HIC: Hospital Infection Control team of our hospital, ASB: endothracheal aspirates, BAL: bronchoalveoar lavage, CVC: central venous device tips, SW: Wound Swab, HAI: Hospital Acquired Infection, SD: Sanitary Direction.

## Competing interests

The authors declare that they have no competition interests.

## Authors' contributions

CFo, MF and CFa, GPT, FL, SN contributed to the conception, review of the studies and data analysis; CFo and MF and SN are also involved in drafting the manuscript. SM and MCB contributed in acquisition and interpretation of data. All authors approved the final version of the manuscript.

## Pre-publication history

The pre-publication history for this paper can be accessed here:


